# Burst Pressure Prediction of Subsea Supercritical CO_2_ Pipelines

**DOI:** 10.3390/ma15103465

**Published:** 2022-05-11

**Authors:** Yan Li, Wen Wang, Zhanfeng Chen, Weipeng Chu, Huijie Wang, He Yang, Chuanyong Wang, Yuxing Li

**Affiliations:** 1 School of Mechanical Engineering, Hangzhou Dianzi University, Hangzhou 310018, China; liyan@wfust.edu.cn (Y.L.); wangwn@hdu.edu.cn (W.W.); c3160109145@163.com (W.C.); whj@hdu.edu.cn (H.W.); yanghe@hdu.edu.cn (H.Y.); wangcy@hdu.edu.cn (C.W.); 2 Facility Horticulture Laboratory of Universities in Shandong, Weifang University of Science and Technology, Weifang 262700, China; 3 Provincial Key Laboratory of Oil and Gas Storage and Transportation Safety in Shandong Province, China University of Petroleum (Huadong), Qingdao 266580, China; liyx@upc.edu.cn

**Keywords:** carbon capture and sequestration (CCS), corrosion defects, unified strength theory, burst pressure

## Abstract

To improve transportation efficiency, a supercritical CO_2_ pipeline is the best choice for large-scale and long-distance transportation inshore and offshore. However, corrosion of the pipe wall will occur as a result of the presence of free water and other impurities present during CO_2_ capture. Defects caused by corrosion can reduce pipe strength and result in pipe failure. In this paper, the burst pressure of subsea supercritical CO_2_ pipelines under high pressure is investigated. First, a mechanical model of corroded CO_2_ pipelines is established. Then, using the unified strength theory (UST), a new burst pressure equation for subsea supercritical CO_2_ pipelines is derived. Next, analysis of the material’s intermediate principal stress parameters is conducted. Lastly, the accuracy of the burst pressure equation of subsea supercritical CO_2_ pipelines is proven to meet the engineering requirement by experimental data. The results indicate that the parameter *b* of UST plays a significant role in determining burst pressure of pipelines. The study can provide a theoretical basis and reference for the design of subsea supercritical CO_2_ pipelines.

## 1. Introduction

**Figure 1:**
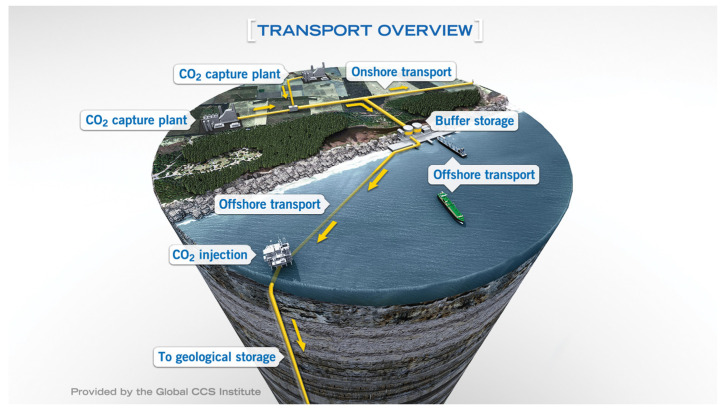
Transportation system in CCS (Source: Provided by Global CCS Institute [8]).

Carbon capture and storage (CCS) technology has become a vital technology for reducing carbon dioxide emissions and improving the climate, as shown in Figure 1 [1]. As part of CCS, CO_2_ is transported through pipelines from the capture point to a suitable geological location. To improve transportation efficiency, high-pressure supercritical CO_2_ transportation is the best choice for inland and offshore transportation of large-scale and long-distance CO_2_. Due to the presence of free water or other corrosive substances that are present in the captured carbon dioxide, corrosion defects will be caused in the pipeline [2]. In addition, corrosion defects can thin the pipe wall and further reduce the pipe’s bearing capacity, which also affects the safe operation of the high-pressure pipes [3,4,5]. As an essential parameter for evaluating pipeline integrity and safety, the burst pressure of the pipeline is usually defined as the ultimate load when the pipeline fails plastically [6]. The accurate prediction of the burst pressure of corroded CO_2_ pipes is necessary for reducing pipeline operation risks and ensuring its strength and safety [7].

Currently, third-party fault assessment models for oil and gas pipes are based primarily on model calculations based on pipeline structural reliability methods, which are not sensitive to pipeline transport media; therefore, the study of the burst pressure of supercritical CO_2_ transportation pipelines can learn from the research methods of high-pressure transportation pipelines. The main research methods are the limitation of state equations based on different criteria, the finite element method [9,10,11], and industry standards, such as ASME [12], DNV [13], CSA [14], PCORRC [15] and other evaluation criteria. In most of the abovementioned methods, the corrosion defects are simplified into geometric shapes containing length and depth to fit the experimental results. Some researchers use neural network to study the failure behavior of pipelines [16,17]; however, empirical models tend to overestimate or underestimate the burst pressure. Hence, some researchers have studied the failure modes of corroded pipelines from a theoretical point of view and proposed some burst pressure equations based on different criteria. Over the last few decades, based on the theory of elastoplastic mechanics, a number of analytical formulas or empirical formulas for the burst pressure of unflawed pipes have been proposed by researchers, and many prediction models for the failure pressure of corroded pipes have been developed. The choice of strength criterion is the key factor for accurately predicting burst pressure, and scholars are also interested in it. Klever et al. [18,19] adopted the Tresca and von Mises yield criteria, considered large strain and material strain hardening, and proposed an analysis model for failure pressure of unflawed pipelines and corrosion-defective pipelines, then verified the analysis by comparison with experimental data. Several studies have demonstrated that the predicted pipe burst pressure is closely correlated with the adopted yield criteria. Christoper et al. [20] conducted experiments to study the burst pressure of unflawed pipes, and found that no single strength criterion could predict the burst pressure of all different types of material. Zhu and Leis [21,22,23] found that the Tresca criterion is appropriate for predicting the burst pressure of high-strain strengthened pipes, whereas the von Mises criterion is suitable for predicting the failure pressure of low-strain strengthened pipes. On this basis, a new multiaxial yield criterion is proposed, namely, the Zhu-Leis criterion, and a theoretical calculation method for the failure pressure of the unflawed pipe was proposed. The theoretical solution has been combined with the results of the pipeline failure pressure experimental data and has a good agreement. Law and Bowie [24] used different criteria to determine the burst pressure of high yield ratio pipelines. It then compared its predictions with the experimental results and determined that every criterion had its own applicability and limitations. Unified strength theory (UST) was first proposed by Yu [25], commonly used in engineering, which takes into account the strength differential effect (SD) of materials and the impact of the intermediate principal stresses of different materials on materials properties. Some researchers have achieved some results when applying UST to the theoretical study of pipeline burst pressure prediction. Based on the von Mises, Tresca, Zhu-Leis criteria, and TS criterion, Wang [26] used the unified strength criterion to derive the failure pressure calculation formula for unflawed thin-walled pipelines. Lin and Deng et al. [27,28,29] proposed the through-walled yield collapse pressure equation of thick-walled pipelines based on UST and verified the accuracy of the equation through experimental data. Zhang [30] proposed a new yield criterion—a weighted unification to predict the burst pressure of a pipe elbow. Deng [31] established a mechanical model capable of calculating the internal pressure strength of metallurgically bonded composite pipes and provided a calculation method for the internal pressure strength. Considering the influence of the ratio of metal yield strength to tensile strength (Y/T) on the bursting pressure, Chen [32] proposed a multi-parameter failure criterion including (Y/T). Chen [33,34] first proposed the DCA model, a theoretical model using thick-walled worn casing, and obtained the stress analytical solution for thick-walled corroded pipes. The model is used to develop a series of burst pressure equations to predict the burst pressure of corroded pipes.

As indicated above, the strength criterion choice in the existing theoretical analysis has a significant effect on the burst pressure of pipelines. Theoretical studies of burst pressures on supercritical CO_2_ transportation pipelines with defects are rare. Due to high pressure design requirements, dense phase or supercritical carbon dioxide requires high wall thickness pipelines [35]; therefore, a novel burst pressure model of corroded dense or supercritical CO_2_ pipelines was proposed based on the DCA model and the UST model. The accuracy of the equations for calculating burst pressure was confirmed with experimental data. An integrity assessment framework is provided by the new equation for the supercritical CO_2_ transportation pipeline. The burst pressure equation is compared with the existing pipeline burst experimental data. The result shows that the error is within the acceptable range of practical engineering applications.

## 2. Unified Strength Theory

The UST is applicable to various materials. The yield criterion of ductile metal materials is a particular form of the strength theory. Generally, ductile metal pipe materials have equal tensile and compressive strengths. The unified strength theory for metallic materials is as follows:

{(1)σ1−11+b(bσ2+σ3)=σUST    σ2≤σ1+σ32(2)11+b (σ1+bσ2)−σ3=σUST   σ2≥σ1+σ32,(3)b=2τs−σtσt−τs           0≤b≤1

The parameter *b* represents the effect of the intermediate principal stress on the material failure. In addition, *b* is a parameter of UST [36]. The UST can be reduced to different strength criteria when the parameter *b* takes different values. For example, the Tresca criterion, the twin-shear stress yield (TS) criterion, the von Mises criterion, and the Zhu-Leis flow theory.

## 3. Mechanical Model of the Corroded Supercritical CO_2_ Pipeline

The unflawed pipeline section is generally two concentric rings, and corrosion defects will cause the pipe wall thickness to be thinned. The types of corrosion defects that have been simplified in the literature are rectangular, parabolic, and point-shaped corrosion [37,38,39,40], but these simplified models make it difficult to conduct theoretical analysis. The theoretical analysis of the wear casing using the double circular arc (DCA) model is shown in Figure 2 in [33]. In this paper, the DCA model is applied to the theoretical analysis of the supercritical CO_2_ pipeline containing corrosion defects. In the model, the corrosion defects are assumed to be long-term corrosion defects, and the pipes with corrosion defects are solved as a plane problem.

**Figure 2:**
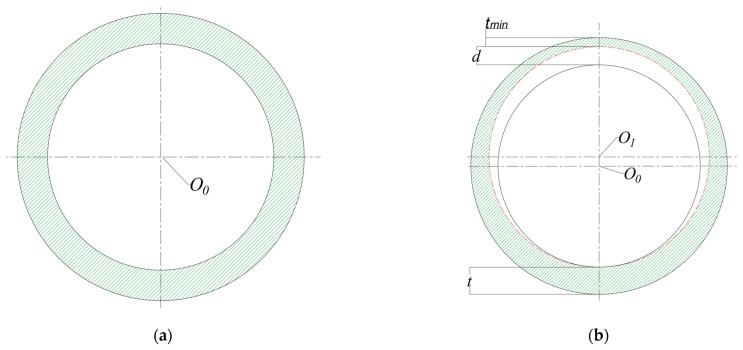
(**a**) Unflawed pipe; (**b**) DCA model of corroded supercritical CO_2_ pipeline.

As shown in Figure 2, Figure 2a shows a cross-sectional view of the unflawed pipe. Figure 2b depicts the cross-section of a pipeline containing corrosion defects. The solid circle is the edge line of the uncorroded pipe, and the dashed circle is the inner edge line of the corroded pipe. *O*_0_ is the initial circle center, and *O*_1_ is the circle center after corrosion. The thickness of the unflawed pipeline wall is *t. t*_min_ is the minimum wall thickness after corrosion. The depth of corrosion defects is *d*. The corrosion ratio of the pipeline is:

(4) $$\varepsilon =\frac{d}{t}$$

(5) $$d=t-t_{\min}$$

## 4. Equation for the Burst Pressure of the Corroded CO_2_ Pipeline

### 4.1. Stress Analysis

**Figure 3:**
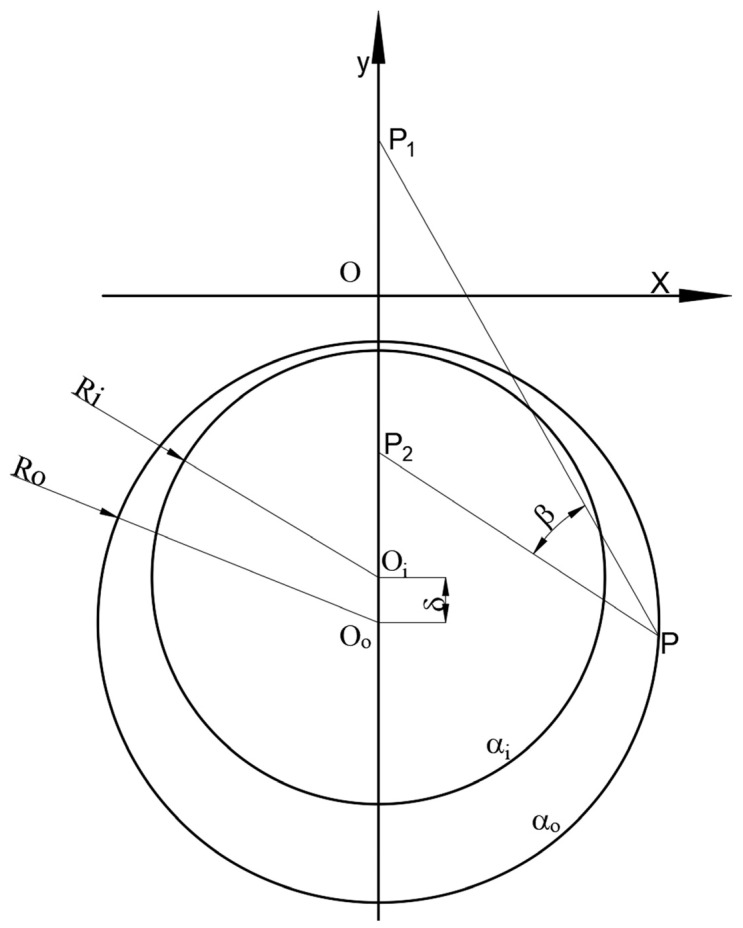
Illustration of the bipolar coordinate system [33].

Figure 3 shows an illustration of the bipolar coordinate system. Based on the DCA model, the stress distribution of the corrosion pipelines under internal pressure can be obtained. An expression for the radial stress of the corroded CO_2_ pipeline is as follows:

(6) $$\begin{array}{l} \sigma_{\alpha }=\frac{1}{2-\cos h2\alpha_{i}-\cos h2\alpha_{0}}\{2p_{i}\sinh^{2}\alpha_{0}-2p_{i}(\cos\beta -\cosh\alpha )\csc h(\alpha_{i}-\alpha_{0})\sinh\alpha_{i}\sinh\alpha_{0}\sinh\alpha \\ -p_{i}\csc h(\alpha_{i}-\alpha_{0})[\sinh(\alpha_{i}+\alpha_{0})-\sinh(\alpha_{i}+\alpha_{0})\cos\beta \cos\alpha -\\ \sinh\left(\alpha_{i}+\alpha_{0}\right)\cosh2\alpha +\sinh\left(\alpha_{i}+\alpha_{0}\right)\cos\beta \cosh3\alpha +\\ 6\cosh\alpha_{i}\cosh\alpha_{0}\cos\beta \sinh\alpha -6\cosh\alpha_{i}\cosh\alpha_{0}\cosh\alpha \sinh\alpha -\\ 4\cosh\left(\alpha_{i}+\alpha_{0}\right)\sinh\alpha \cos\beta -2\cosh\left(\alpha_{i}+\alpha_{0}\right)\sinh\alpha \cosh2\alpha \cos\beta +\\ 3\cosh\left(\alpha_{i}+\alpha_{0}\right)\sinh2\alpha ]\} \end{array}$$

The hoop stress of the corroded supercritical CO_2_ pipelines can be expressed as:

(7) $$\begin{array}{l} \sigma_{\beta }=\frac{1}{2-\cos h2\alpha_{i}-\cosh2\alpha_{0}}\{2p_{i}\sinh^{2}\alpha_{0}+2p_{i}(\cos\beta -\cosh\alpha )\csc h(\alpha_{i}-\alpha_{0})\sinh\alpha_{i}\sinh\alpha_{0}\sinh\alpha -\\ p_{i}\csc h(\alpha_{i}-\alpha_{0})[\sinh(\alpha_{i}+\alpha_{0})-3\sin h(\alpha_{i}+\alpha_{0})\cos\alpha \cos\beta +\\ \sinh(\alpha_{i}+\alpha_{0})\cosh2\alpha +2\sin(\alpha_{i}+\alpha_{0})\cosh2\alpha \cos2\beta -\\ \sinh(\alpha_{i}+\alpha_{0})\cosh3\alpha \cos\beta -6\cosh\alpha_{i}\cosh\alpha_{0}\sinh\alpha \cos\beta +\\ 6\cosh\alpha_{i}\cosh\alpha_{0}\sinh\alpha \cos\alpha +8\cosh(\alpha_{i}+\alpha_{0})\sinh\alpha \cos\beta +\\ 2\cosh(\alpha_{i}+\alpha_{0})\sinh\alpha \cosh2\alpha \cos\beta -3\cosh(\alpha_{i}+\alpha_{0})\sinh2\alpha -\\ 2\cosh(\alpha_{i}+\alpha_{0})\sinh2\alpha \cos2\beta ]\} \end{array}$$

The shear stress of the corroded supercritical CO_2_ pipeline can be expressed as:

(8) $$\tau_{\alpha \beta }=-\frac{4p_{i}\csc h(\alpha_{i}-\alpha_{o})\sinh(\alpha_{i}-\alpha )(\cosh\alpha -\cos\beta )\sin\beta }{2-\cosh2\alpha_{i}-\cosh2\alpha_{o}}$$

Substituting $\alpha =\alpha_{i}$ and β=π into Equation (6), the radial stress of the corroded supercritical CO_2_ pipeline can be simplified as:

(9) $$\sigma_{\alpha }=-p_{i}$$

Substituting $\alpha =\alpha_{i}$ and β=π into Equation (7), the hoop stress of the corroded supercritical CO_2_ pipeline can be expressed as:

(10) $$\sigma_{\beta }=\frac{p_{i}(1-q^{2}+\frac{2q^{2}(q+\sqrt{f^{2}+q^{2}}}{f^{2}(\sqrt{1+f^{2}-\sqrt{f^{2}+q^{2}}}})}{1+q^{2}},$$

where $f=\frac{\sqrt{q^{4}+{\left(-1+k^{2}\right)}^{2}-2q^{2}(1+k^{2})}}{2k}$;

*q* is an intermediate variable and q=1−2/ξ;
*k* is an intermediate variable and k=2ε/ξ.

Simplifying Equation (10), we have the hoop stress of pipes with corrosion defects:

(11) $$\sigma_{\beta }=\frac{p_{i}[1+q^{4}-2qk+2q^{3}k-k^{2}+q^{2}(2+k^{2})]}{\left(1+q^{2})(-1+q^{2}+2qk+k^{2}\right)}$$

Substituting *q* and *k* into Equation (11), the hoop stress of the supercritical CO_2_ pipelines with corrosion defects can be further expressed as:

(12) $$\sigma_{\beta }=\frac{p_{i}[4\varepsilon^{2}(-1+\xi )+4\varepsilon (2-3\xi +\xi^{2})-(2-2\xi +\xi^{2})]}{2(-1+\varepsilon )(-1+\varepsilon +\xi )(2-2\xi +\xi^{2})}$$

where ξ=t/D.

For the DCA model, the max stress is hoop stress $\sigma_{\beta }$, the axial stress is $\sigma_{z}=\mu (\sigma_{\alpha }-p_{i})$, so $\sigma_{z}$ can be obtained:

(13) $$\sigma_{z}=p_{i}\left\{-0.3+\frac{0.15\left[4\varepsilon^{2}(-1+\xi )+4\varepsilon \left(2-3\xi +\xi^{2}\right)-{\left(2-2\xi +\xi^{2}\right)}^{2}\right]}{(-1+\varepsilon )(-1+\varepsilon +\xi )\left(2-2\xi +\xi^{2}\right)}\right\}$$

We have three principal stresses of the supercritical CO_2_ pipelines with corrosion defects:

(14) $$\left\{ \begin{array}{l} \sigma_{1}=\sigma_{\beta }\\ \sigma_{2}=\sigma_{z}\\ \sigma_{3}=\sigma_{\alpha } \end{array}\right.$$

### 4.2. Determination of Burst Pressure Equation Based on UST

To determine the failure pressure of corroded pipelines, the strain failure criterion and the stress failure criterion are the most commonly employed [41]. Experimental evidence shows that the stress failure criterion is highly accurate when applied to calculate the burst pressure of corroded pipelines. According to this section, we use the stress failure criterion, which means that the pipeline fails as soon as the effective stress of part of the corrosion reaches the tensile strength of the pipe. Substituting Equation (14) into Equation (1), a novel prediction equation can be used for the corroded CO_2_ pipelines to predict the burst pressure as follows:

(15) $$P_{b}=\frac{\left(1+b)(f_{0}+f_{1}\varepsilon^{2}+f_{2}\varepsilon \right)}{\omega_{0}+b\omega_{1}+\varepsilon^{2}(\omega_{2}+b\omega_{3})+\varepsilon (\omega_{4}+b\omega_{5})}\sigma_{u}$$

where

$$\begin{array}{l} f_{0}=2-4\lambda +3\lambda^{2}-\lambda^{3}\\ f_{1}=2-2\lambda +\lambda^{2}\\ f_{2}=-4+6\lambda -4\lambda^{2}+\lambda^{3}\\ \omega_{0}=-\lambda^{2}+\lambda^{3}-0.5\lambda^{4}\\ \omega_{1}=-0.8+1.6\lambda -1.9\lambda^{2}+1.1\lambda^{3}-0.35\lambda^{4}\\ \omega_{2}=\lambda^{2}\\ \omega_{3}=-0.8+0.8\lambda +0.3\lambda^{2}\\ \omega_{4}=-2\lambda^{2}+\lambda^{3}\\ \omega_{5}=1.6-2.4\lambda +0.2\lambda^{2}+0.3\lambda^{3}\\ \lambda =D/t \end{array}$$

Equation (15) is the equation of the corroded CO_2_ pipeline based on the UST. Equation (15) is a function of the geometric parameters of the pipeline (*D* and *t*), the geometric parameters of the corrosion defect (*d*), and the pipeline material characteristics (parameter *b*). It is not a single equation for predicting burst pressure of pipelines but a series equation under different strength criteria when parameter *b* takes different values. For the supercritical CO_2_ pipeline without defects (ε=0), the burst pressure equation can be simplified as:

(16) $$P_{b}=\frac{f_{0}(1+b)}{\omega_{0}+b\omega_{1}}\sigma_{u}$$

### 4.3. Equations of Burst Pressure under Different Yield Criteria

The material parameter *b* has significant impact on the ultimate burst pressure. As a bridge between UST and different strength criteria, different *b* values are associated with different strength criteria, such as TS, Tresca, von Mises, and the Zhu-Leis flow theory; therefore, the burst pressure equation for a variety of materials pipe can be obtained.

#### 4.3.1. The Burst Pressure Equation Based Tresca Criterion

The Tresca criterion can be deduced from UST when the parameter *b* = 0 [42], the burst pressure equation of CO_2_ pipelines with or without defects can be presented by solving Equations (15) and (16):

(17) $$P_{b\text{-}\mathrm{with}}^{T}=\frac{f_{0}+f_{1}\varepsilon^{2}+f_{2}\varepsilon }{\omega_{0}+\varepsilon^{2}\omega_{2}+\varepsilon \omega_{4}}\sigma_{u}$$

(18) $$P_{b\text{-}\mathrm{without}}^{T}=\frac{f_{0}}{\omega_{0}}\sigma_{u}$$

#### 4.3.2. The Burst Pressure Equation Based on the on Mises Criterion

The von Mises criteria can be derived when the parameter $b=1/(1+\sqrt{3})$ [42], and the burst pressure equation of pipelines with or without defects can be presented by solving Equations (15) and (16):

(19) $$P_{b\text{-}\mathrm{with}}^{M}=\frac{\left(2+\sqrt{3})(f_{0}+f_{1}\varepsilon^{2}+f_{2}\varepsilon \right)}{\left(1+\sqrt{3})[\omega_{0}+b\omega_{1}+\varepsilon^{2}(\omega_{2}+b\omega_{3})+\varepsilon (\omega_{4}+b\omega_{5})\right]}\sigma_{u}$$

(20) $$P_{b\text{-}\mathrm{without}}^{M}=\frac{(2+\sqrt{3})f_{0}}{\left(1+\sqrt{3})(\omega_{0}+b\omega_{1}\right)}\sigma_{u}$$

#### 4.3.3. The Burst Pressure Equation Based on the Zhu-Leis Flow Theory

The Zhu-Leis flow theory can be deduced when the parameter $b=(1+\sqrt{3})/16$ [27], and the burst pressure equation of pipelines with or without defects can be presented by solving Equations (15) and (16):

(21) $$P_{b\text{-}\mathrm{with}}^{Z}=\frac{\left(17+\sqrt{3})(f_{0}+f_{1}\varepsilon^{2}+f_{2}\varepsilon \right)}{16[\omega_{0}+b\omega_{1}+\varepsilon^{2}(\omega_{2}+b\omega_{3})+\varepsilon (\omega_{4}+b\omega_{5})]}\sigma_{u}$$

(22) $$P_{b\text{-}\mathrm{without}}^{Z}=\frac{f_{0}(17+\sqrt{3})}{16(\omega_{0}+b\omega_{1})}\sigma_{u}$$

#### 4.3.4. The Burst Pressure Equation Based on the TS Criterion

The TS criterion can be deduced when the parameter b=1, and the burst pressure equation of pipelines with or without defects can be presented by solving Equations (15) and (16):

(23) $$P_{b\text{-}\mathrm{with}}^{TS}=\frac{2(f_{0}+f_{1}\varepsilon^{2}+f_{2}\varepsilon )}{\omega_{0}+b\omega_{1}+\varepsilon^{2}(\omega_{2}+b\omega_{3})+\varepsilon (\omega_{4}+b\omega_{5})}\sigma_{u}$$

(24) $$P_{b\text{-}\mathrm{without}}^{TS}=\frac{2f_{0}}{\omega_{0}+b\omega_{1}}\sigma_{u}$$

## 5. Influence of Parameter *b* on Burst Pressure

**Figure 4:**
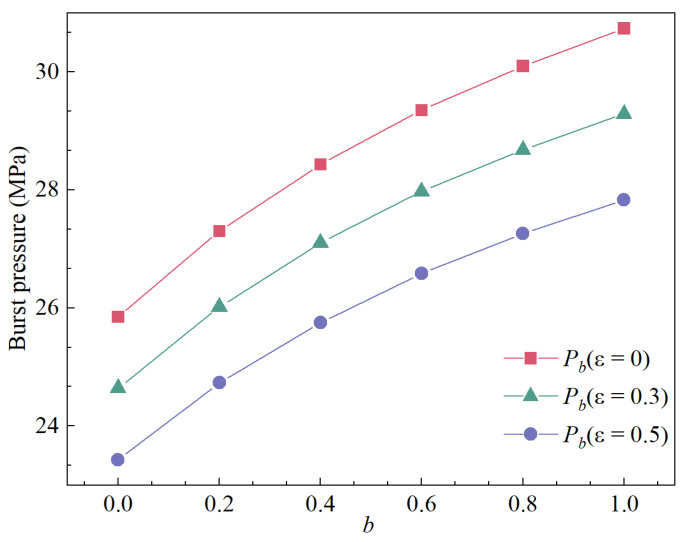
The influence of parameter *b* on burst pressure.

**Figure 5:**
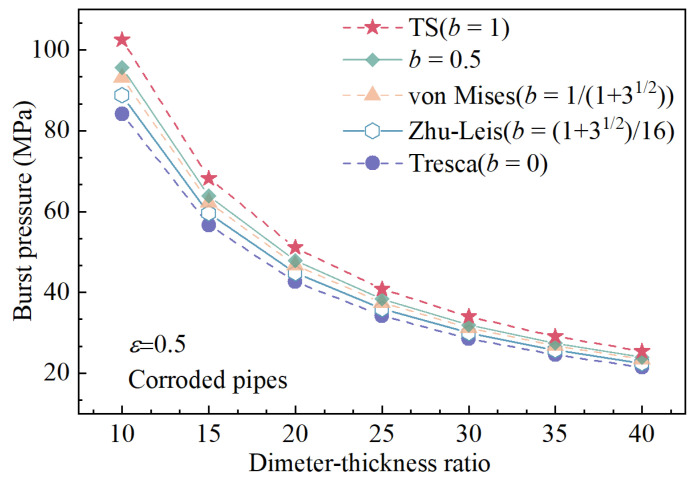
The influence of diameter–thickness ratio on burst pressure.

In this section, X65 grade steel, commonly used for CO_2_ pipeline transportation [43], was selected to investigate the effect of parameter *b* on the burst pressure. The material properties and geometric parameters are listed in Table 1 [44]. The burst pressure of corroded pipelines of API X65 at different values of parameter *b* and different corrosion rates were calculated by using Equation (15). The results have been shown in Figure 4 and Figure 5.

Figure 4 and Figure 5 indicated that the influence of *b* on the burst pressure is significant and cannot be ignored. Figure 4 shows that the trend of burst pressure for different corrosion rates is consistent for increasing values of parameter *b*; therefore, proper selection of parameter *b* is the key to accurately predicting burst pressure. As shown in Figure 5, equations of different criteria divided from Equation (15) have been used to calculate the burst pressure when the corrosion ratio is 0.5 [28]. It can be seen that as the pipe diameter-thickness ratio increases, the pipe burst pressure drops rapidly, and the downward trend gradually slows down as the pipe diameter–thickness ratio increases. In addition, the yield criteria under different *b* values have a great influence on the burst pressure. The result indicates that when *b* = 0 (Tresca), the calculated value is the lower limit of predictive burst pressure while *b* = 1 (TS) the upper limit.

## 6. Validations and Discussions

In this section, the equation of the corroded pipeline suggested in this paper will be verified by comparing it with experimental data of pipeline burst pressure reported in the literature [45]. In the following evaluation, two important statistical error parameters are used, one is the average error (average relative error), and the other is the standard deviation of the average error. These two parameters are defined as follows [23].

(25) $$\mathrm{Mean}\;\mathrm{error}(\mathrm{ME})=\frac{\sum \left(P_{i}^{cal}/P_{i}^{\exp}-1\right)}{N}$$

(26) $$\mathrm{Standard}\;\mathrm{deviation}(\mathrm{SD})=\sqrt{\frac{\sum {\left(P_{i}^{cal}/P_{i}^{\exp}-1-ME\right)}^{2}}{N-1}}$$

### 6.1. Comparisons with Experimental Data for Unflawed Pipeline

According to the abovementioned analysis, only when parameter *b* is determined can the burst pressure of the pipeline can be calculated. Generally, once the shear and tensile strength are obtained from the material experiment, the value of parameter b can be calculated by Equation (3). The tensile strength is easy to get by experiment, however, the shear strength is not easy to obtain directly. Hence, the same *b* value (*b* = 0.5) which was used for the thick-walled tube to predict the through-wall yield ductile pressure in the literature [28], is used in this paper to calculate the burst pressure of unflawed pipelines to verify the accuracy of the proposed unflawed burst pressure equation. Thirty-two sets of full-scale experimental data of unflawed pipes were collected from the literature [45]. The range of the diameter–thickness ratio of the experimental sample is from 5 to 50, including both thick-walled pipes and thin-walled pipes. The comparative results are summarized in Table 2. $P_{Equation\;(16)}$ denotes the burst pressure calculated using Equation (16).

**Figure 6:**
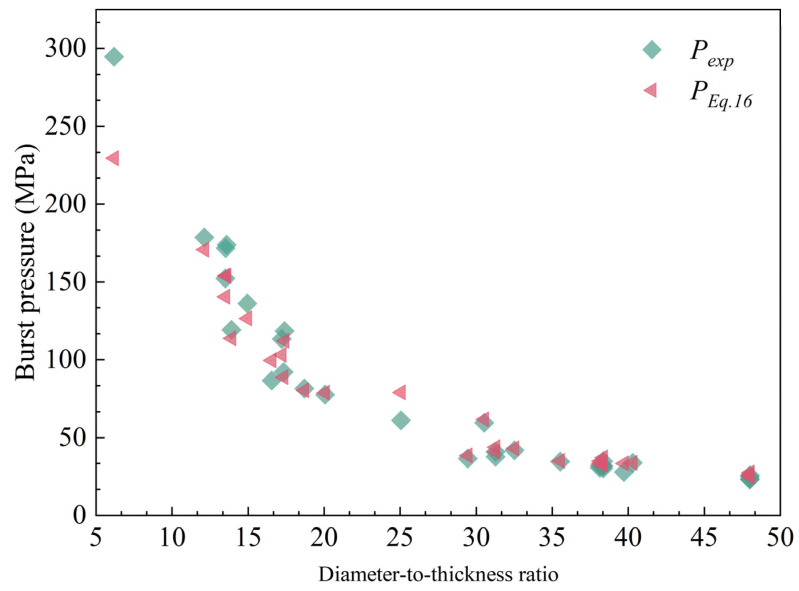
Comparison of predictions with experiments.

Table 2 indicated that the average is 1.03, and the calculated results have a good agreement with the experiments. By analyzing the above data, excluding two samples (No. 19, No. 32) of experimental samples with large errors, the relative errors are within 20%. The ME is 6.9%. The large errors are considered to have occurred due to data collection errors in the process of the experiment or due to the value of *b*. The experiments and the predicted results of unflawed pipes are also shown in Figure 6. As the diameter–thickness ratio increases, the predicted burst pressure is closer to the experimental value. The influence of parameter *b* on the burst pressure of pipelines may be related to the pipeline material and diameter-thickness ratio, which is not particularly clear at present and needs to be further studied.

### 6.2. Comparisons with Experimental Data for Corroded Pipelines

In this section, a comparison of the predicted burst pressure of corroded supercritical CO_2_ pipes with existing experimental data [46] is presented. The calculated results by Equation (15) and comparison results are listed in Appendix A. The corrosion rate of the experimental samples in the literature ranges from 0.02 to 0.83, and the ratio of diameter to wall thickness ranges from 16 to 88. Detailed geometric parameters and material properties of corroded pipelines are shown in Appendix A. According to the previous analysis of unflawed pipelines, when verifying the burst pressure equation of corroded pipes with, the value of parameter *b*, it is 0.5 [28].

**Figure 7:**
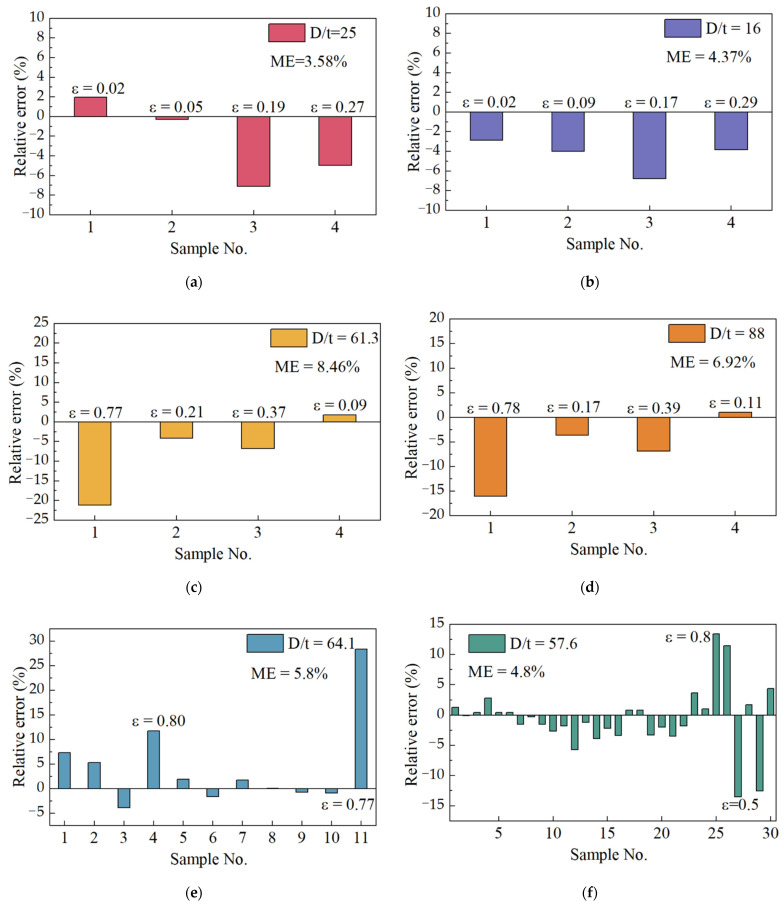
Errors between predictions and experiments at different corrosion rate.

To verify the accuracy of the model under a different corrosion ratio, the experimental data in the literature are grouped [46]. According to the diameter–thickness ratio, it is divided into six groups to verify the accuracy of this model. The experimental specimens contain one group of thick-walled tubes (*D*/*t* < 20) and five groups of thick-walled tubes (*D*/*t* > 20). The comparison results are shown in Figure 7. As can be seen in Figure 7, the errors of the model are within the engineering requirements for different diameter-thickness ratios. Moreover, the corrosion ratio for each experimental sample in Figure 7a–d have been labeled in the figures. In Figure 7e,f, due to the limited space of the figure, only the corrosion rate of the sample with the largest error in the predicted value is marked, and the specific corrosion situation can be found in Appendix A. It is not difficult to see, that when the corrosion ratio in 0.5–0.8, the predicted value of the error is larger. When the corrosion ratio is below 0.5, the burst pressure prediction model proposed in this paper has a high accuracy and the error is within 10%.

To demonstrate the advantages of our model, the prediction calculated using Chen’s equation [47] and RAM PIPE are also compared with the model in this paper. As shown in Table 3, ME is 4.57%, 6.7%, and 18.1%, and SD is 0.055, 0.062, and 0.084. The calculated results using Equation (15) are more accurate than Chen’s equation and RAM PIPE. The results indicate that the burst pressure equation proposed in this paper has a good consistency and low dispersion.

## 7. Conclusions

Based on the UST and DCA model, a unified burst pressure equation for a supercritical CO_2_ transport pipeline has been proposed, and the effect of intermediate principal stress on the predicted burst pressure was considered. The bursting pressure increases with the increase of the *b* value, and it decreases with the increase of the diameter–thickness ratio. Through the discussion, it is evident that different yield criteria based on the unified strength theory have a significant impact on the accuracy of prediction burst pressure. Finally, the accuracy of the predicted burst pressure equations in this paper have been verified by comparison with the experimental data, and the results indicate that our equations are reasonably accurate, especially when the corrosion rate is below 0.5. The findings of this research can provide a theoretical basis for the transportation and storage of carbon dioxide; however, the material intermediate principle, stress parameter *b*, is not sufficiently understood. It is necessary to further study how to determine the value of *b* in specific applications.

## Figures and Tables

**Table 1:** The material properties and geometrical parameters of API 5L X65 (Date from literature [44].

Parameters	Value
Steel grade	X65
Yield strength ($\sigma_{y}$/MPa)	467
Ultimate tensile strength ($\sigma_{u}$/MPa)	576
Diameter (mm)	762
Wall-thickness (mm)	17.5

**Table 2:** Comparisons between the calculation results and the experiments.

No.	*D* (mm)	*t* (mm)	$\sigma_{y}\;(\mathrm{MPa})\;$	$\sigma_{u}\;(\mathrm{MPa})\;$	$P_{\exp}\;(\mathrm{MPa})\;$	$P_{Equation(16)}\;(\mathrm{MPa})\;$	$P_{E\mathrm{quation}(16)}/P_{\exp}$
1	912	19	457.8	546.0	23.11	24.90	1.08
2	912	19	426.7	578.0	23.17	26.36	1.14
3	912	19	517.1	559.0	24.85	25.49	1.03
4	912	19	508.8	604.0	25.80	27.55	1.07
5	893.7	22.5	526.0	608.0	27.93	33.40	1.20
6	609.6	15.9	501.2	581.0	30.20	33.05	1.09
7	762.4	20	531.5	608.0	30.63	34.78	1.14
8	609.6	15.9	511.5	600.0	31.72	34.13	1.08
9	609.6	15.9	440.5	585.0	31.76	33.27	1.05
10	762.4	20	555.0	580.0	31.95	33.18	1.04
11	544.05	13.5	623.9	624.0	33.84	33.80	1.00
12	507.93	14.3	508.8	571.0	34.50	35.00	1.01
13	609.6	15.9	534.3	653.0	34.79	37.14	1.07
14	397.6	13.5	364.0	523.0	36.50	38.50	1.05
15	591.2	18.9	563.0	589.0	37.68	40.88	1.08
16	591.2	18.9	607.0	630.0	40.79	43.73	1.07
17	591.8	18.2	636.0	645.0	41.76	43.11	1.03
18	390.8	12.8	807.0	869.0	59.60	61.76	1.04
19	247.1	9.86	641.1	916.9	61.08	78.96	1.29
20	179.4	8.94	468.8	737.7	77.70	78.73	1.01
21	252.4	13.5	606.7	703.2	81.56	80.32	0.98
22	162.2	9.8	602.0	776.0	86.60	99.57	1.15
23	180.3	10.4	613.6	723.8	92.17	88.86	0.96
24	67.3	3.91	689.4	834.2	113.34	103.12	0.91
25	179.1	10.3	848.0	916.9	118.51	112.24	0.95
26	90.35	6.5	696.3	751.4	119.27	113.74	0.95
27	179.6	12.01	779.0	896.2	136.09	126.61	0.93
28	179.5	13.3	834.2	903.1	152.29	140.54	0.92
29	198.9	14.7	903.1	992.7	171.66	154.11	0.90
30	198.2	14.6	903.1	992.7	173.80	153.64	0.88
31	180.6	14.9	903.1	992.7	178.55	170.82	0.96
32	89	14.4	606.7	730.8	294.65	229.47	0.78
						Mean	1.03

**Table 3:** Comparison with experimental data and other models.

Comparison Results	*P_Equation_*_(15)_	*P_Chen_*	*P_RAM_*
ME	4.57%	6.7%	18.1%
SD	0.055	0.062	0.084

## Nomenclature

$\sigma_{1},\sigma_{2},\sigma_{3}$	First principal stress, second principal stress, third principal stress
$\sigma_{\alpha },\sigma_{\beta },\sigma_{z}$	Radial stress, hoop stress, axial stress
$f_{0},f_{1},f_{2}$	Coefficients in Equations (15) and (16)
$\omega_{0},\cdot \cdot \cdot ,\omega_{5}$	Coefficients in Equations (15) and (16)
$\sigma_{\mathrm{UST}}$	UST equivalent stress
$\tau_{s},\tau_{\alpha \beta }$	Shear strength and shear stress
a	Yield-to-tensile strength ratio
t	Influence coefficient of intermediate principal stress on material failure
	Thickness of an ideal pipeline
$t_{\min}$	Minimum wall thickness after corrosion
d	Depth of corrosion defect
ε	Corrosion ratio
$p_{i},p_{b}$	Inner pressure, the burst pressure of the pipeline
λ	The ratio of thickness to diameter
μ	Poisson’s ratio
$\sigma_{u},\sigma_{y}$	Ultimate tensile strength, yield strength
α,β	Variables in bipolar coordinate system
$P_{\exp}$	Experimental bursting pressure
q, k	Intermediate variable
$\sigma_{t}$	Tensile strength
$P_{i}^{cal}$	The calculated burst pressure using Equation (15) in Table 2 and Appendix A
$P_{i}^{\exp}$	The experimental data of burst pressure for the i-th sample
N	The total number of experiments
$P_{Chen}$	Burst pressure calculated by Chen’s model.

## Appendix A

**Table A1:** Errors of predictions compared with the actual burst pressure and two other evaluation model. (Experimental data source [46]).

No.	*D* (mm)	*t* (mm)	*σ_y_* (MPa)	*σ_u_* (MPa)	*d* (mm)	*P_exp_* (MPa)	*P_Equation_*_(15)_ (MPa)	Errors *(P_Equation_*_(15)_)	Errors (*P_Chen_*)	Errors (*P_RAM_*)
1	342	13.5	840	980	0.24	80.6	82.19	1.97%	6.45%	0.74%
2	342	13.5	840	980	0.64	80.2	79.97	−0.29%	3.87%	−5.99%
3	342	13.5	840	980	2.54	74.5	69.22	−7.09%	−4.03%	−22.01%
4	342	13.5	840	980	3.64	66.1	62.85	−4.92%	−2.27%	−23.90%
5	252	15.7	930	1070	0.33	143	138.9	−2.87%	5.94%	−2.66%
6	252	15.7	930	1070	1.43	136	130.57	−3.99%	4.04%	−13.60%
7	252	15.7	930	1070	2.63	130	121.23	−6.75%	0.31%	−22.08%
8	252	15.7	930	1070	4.53	110	105.91	−3.72%	2.36%	−26.45%
9	1219	19.9	585	715	15.41	7.6	5.99	−21.18%	−25.00%	−40.79%
10	1219	19.9	585	715	4.12	21.4	20.52	−4.11%	−7.01%	−16.82%
11	1219	19.9	592	723	7.44	17.7	16.51	−6.72%	−9.60%	−23.73%
12	1219	19.9	592	723	1.77	23.3	23.71	1.76%	−0.86%	−6.87%
13	1219	13.8	568	705	10.78	4.7	3.94	−16.17%	−21.28%	−34.04%
14	1219	13.8	568	705	2.3	15.3	14.74	−3.66%	−7.84%	−13.73%
15	1219	13.8	589	731	5.45	12	11.17	−6.92%	−10.83%	−21.67%
16	1219	13.8	589	731	1.54	16.1	16.27	1.06%	−3.11%	−8.07%
17	1320	22.9	782	803	2.52	27	27.35	1.30%	7.04%	−8.52%
18	1320	22.9	782	803	2.27	27.7	27.67	−0.11%	5.78%	−9.03%
19	1320	22.9	782	803	2.31	27.5	27.62	0.44%	6.18%	−8.73%
20	1320	22.9	782	803	6.73	21.3	21.89	2.77%	7.98%	−14.08%
21	1320	22.9	782	803	6.73	21.8	21.89	0.41%	5.50%	−16.06%
22	1320	22.9	782	803	6.57	22	22.1	0.45%	5.45%	−15.91%
23	1320	22.9	782	803	11.45	15.9	15.66	−1.51%	3.14%	−22.64%
24	1320	22.9	782	803	11.45	15.7	15.66	−0.25%	4.46%	−21.66%
25	1320	22.9	782	803	11.45	15.9	15.66	−1.51%	3.14%	−22.64%
26	1320	22.9	782	803	18.55	6.2	6.04	−2.58%	1.61%	−29.03%
27	1320	22.9	782	803	19.01	5.5	5.41	−1.64%	1.82%	−27.27%
28	1320	22.9	782	803	18.55	6.4	6.04	−5.63%	−1.56%	−31.25%
29	1320	20.6	782	803	2.06	23.2	24.89	7.28%	13.36%	−2.16%
30	1320	20.6	782	803	5.89	18.9	19.91	5.34%	10.58%	−11.11%
31	1320	20.6	782	803	11.33	13.2	12.69	−3.86%	0.00%	−24.24%
32	1320	20.6	782	803	16.48	5.1	5.7	11.76%	15.69%	−15.69%
33	1320	22.9	782	803	4.58	25	24.69	−1.24%	4.00%	−14.40%
34	1320	22.9	782	803	4.58	25.7	24.69	−3.93%	1.17%	−16.73%
35	1320	22.9	782	803	11.45	16	15.66	−2.13%	2.50%	−23.13%
36	1320	22.9	782	803	11.45	16.2	15.66	−3.33%	1.23%	−24.07%
37	1320	22.9	782	803	18.32	6.3	6.36	0.95%	4.76%	−25.40%
38	1320	22.9	782	803	18.32	6.3	6.36	0.95%	4.76%	−25.40%
39	1320	20.6	782	803	4.12	21.8	22.22	1.93%	7.34%	−11.01%
40	1320	20.6	782	803	10.3	14.3	14.07	−1.61%	2.80%	−21.68%
41	1320	20.6	782	803	16.85	5.1	5.19	1.76%	5.88%	−23.53%
42	1320	22.9	782	803	2.29	28.6	27.65	−3.32%	2.10%	−12.24%
43	1320	22.9	782	803	2.29	28.2	27.65	−1.95%	3.55%	−10.99%
44	1320	22.9	782	803	6.87	22.5	21.71	−3.51%	1.33%	−19.56%
45	1320	22.9	782	803	6.87	22.1	22.71	2.76%	3.17%	−18.10%
46	1320	22.9	782	803	11.45	15.1	15.66	3.71%	8.61%	−18.54%
47	1320	22.9	782	803	11.45	15.5	15.66	1.03%	5.81%	−20.65%
48	1320	22.9	782	803	18.32	5.6	6.36	13.57%	17.86%	−16.07%
49	1320	22.9	782	803	18.32	5.7	6.36	11.58%	15.79%	−17.54%
50	1320	20.6	782	803	2.27	24.6	24.62	0.08%	5.69%	−9.35%
51	1320	20.6	782	803	6.39	19.4	19.25	−0.77%	4.12%	−16.49%
52	1320	20.6	782	803	10.3	14.2	14.07	−0.92%	3.52%	−21.13%
53	1320	20.6	782	803	15.86	5.1	6.55	28.43%	33.33%	−3.92%
54	1320	22.9	782	803	11.45	18.1	15.66	−13.48%	−9.39%	−32.04%
55	1320	22.9	782	803	11.45	15.4	15.66	1.69%	6.49%	−20.13%
56	1320	22.9	782	803	11.45	17.9	15.66	−12.51%	−8.38%	−31.28%
57	1320	22.9	782	803	11.45	15	15.66	4.40%	9.33%	−18.00%
